# When color helps

**DOI:** 10.31744/einstein_journal/2019AO4410

**Published:** 2018-12-17

**Authors:** Ana Luiza Fontes de Azevedo Costa, Thiago Gonçalves dos Santos Martins, Ricardo Vieira Martins, Paulo Schor

**Affiliations:** 1 Hospital das Clínicas, Faculdade de Medicina, Universidade de São Paulo, São Paulo, SP, Brasil.; 2 Universidade Federal de São Paulo, São Paulo, SP, Brasil.; 3 Universidade Federal do Rio de Janeiro, Rio de Janeiro, RJ, Brasil.; 4 Escola Paulista de Medicina, Universidade Federal de São Paulo, São Paulo, SP, Brasil.

**Keywords:** Ophthalmic solutions, Patient safety, Color, Consumer behavior, Drug labeling/methods, Drug packaging/methods, Soluções oftálmicas, Segurança do paciente, Cor, Comportamento do consumidor, Rotulagem de medicamentos/métodos, Embalagem de medicamentos/métodos

## Abstract

**Objective:**

To reduce the inappropriate identification of eye drops, through the use of different colors.

**Methods:**

A group of 34 healthy volunteers was presented to two groups of four eye drops each. All eye drops were placed in identical, unlabelled vials. In one group, all four eye drops were transparent. In the other group, each had a different color. A number was assigned to each eye drop, and the volunteer was asked to identify it by color. We measured the correct index in the identification of the eye drops of the two groups.

**Results:**

The volunteers had a level of education from incomplete junior school to complete graduate course, with 16 males (48%) and 18 females (52%), age range of 21 to 87 years. The success rate in the group of colored eye drops was 88% and, in the group of transparent, 24%.

**Conclusion:**

The use of colorings in eye drops can help distinguishing the vials and preventing misidentification.

## RESUMO

**Objetivo:**

Reduzir a identificação errônea de colírios, por meio do uso de substâncias com cores diferentes.

**Métodos:**

Um grupo de 34 voluntários saudáveis foi apresentado a dois grupos de quatro colírios cada. Todos os colírios foram colocados em frascos idênticos sem rótulo. Em um grupo de quatro colírios, todos tinham conteúdos transparentes. No outro grupo, cada um dos quatro possuía uma substância de cor diferente. A cada um foi atribuído um número, e o voluntário foi solicitado a identificá-lo por meio da cor. Medimos o índice de acerto na identificação dos colírios dos dois grupos.

**Resultados:**

Os voluntários possuíam nível de formação desde Fundamental incompleto até Pós-Graduação completa, sendo 16 do sexo masculino (48%) e 18 do sexo feminino (52%), com idades variando de 21 até 87 anos. O índice de acerto no grupo de colírios coloridos foi de 88% e, no grupo de colírios transparentes, de 24%.

**Conclusão:**

O uso de colorações em colírios pode auxiliar na diferenciação entre os frascos e prevenir a identificação errônea.

## INTRODUCTION

Over the life span of evolution, the retinal photoreceptors were modified to allow the survival of mammals, and this changed their habits from nighttime to daytime. Human beings have three types of cones, which are photoreceptors capable of absorbing different wavelengths: short, medium, and long (blue, green, and red, respectively).^(^ ^1^ ^)^

Human beings are capable of distinguishing approximately 16 million colors. This perception depends on the activation of the cones at different intensities. The vision of colors is an important resource, which allowed humans to better evaluate all the complexity of the environment. Additionally, it contributed to the memorization of visual scenes.^(^ ^2^ ^)^

Considering the importance of colors in identifying the environment, we noted that they could be a useful attribute to help differentiating among the various types of eye-drops that are currently transparent and have similar packaging.

The incorrect use of eye-drops is a problem faced by numerous patients. Oftentimes, it is poorly recognized by patients, who are not aware of the error, and by healthcare professionals, who have not control over the form patients use eyedrops in everyday life.

When evaluating eye-drop flasks available in the market, we observe that eye-drops from the same company have very similar recipients. Even with intact vision, the switch over of eye-drops can occur, even with systemic use drugs that are dispensed in drops. Even when the lids have different colors, there can be an exchange of the lids, resulting in incorrect use.^(^ ^3^ ^,^ ^4^ ^)^

## OBJECTIVE

To reduce incorrect identification of eye-drops by using different colors to identify them.

## METHODS

A clinical trial with a randomized sample was carried out. Thirty four participants were selected randomly. The previous ophthalmologic examination was done, and those who had any impairment of visual acuity or of color perception were excluded.

The study protocol was approved by the Research Ethics Committee of the *Faculdade de Medicina da Universidade Federal de São Paulo*, under protocol no. 2.408.088, CAAE: 60480115.7.0000.5505.

**Figure 1:**
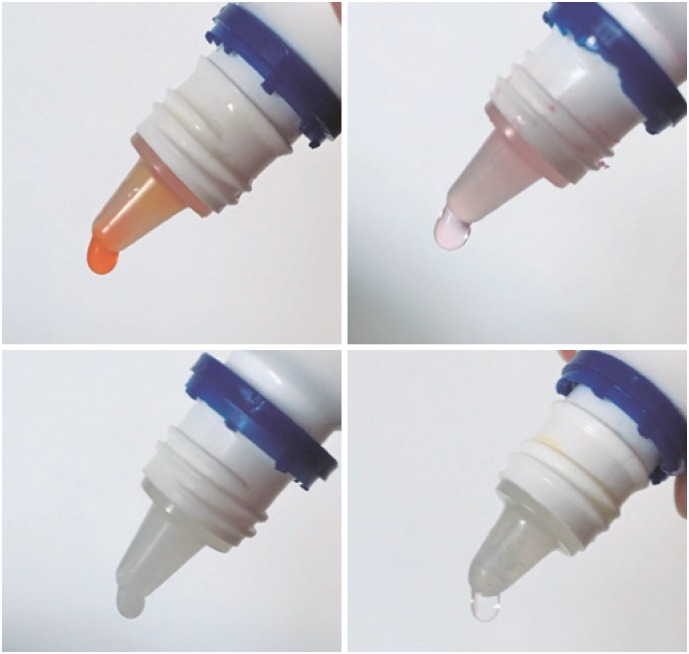
Eye-drops with substances of different colors utilized in the identification test

The 34 volunteers were presented with eight eye-drops in two groups of four eye-drops each (Figure 1). The eye-drops were in identical bottles, but contained different substances. The first group of eye-drops had substances of different colors (yellow colored 1% sodium fluorescein; one had artificial tears containing rose-toned vitamin B12; white colored 1% prednisolone acetate; and transparent 0.1% brimonidine tartrate). The second group of eye-drops was composed of transparent substances (5mg/mL carmellose sodium, 0.25mg/mL benzylisoquinolinium fumarate, 10mg/mL tropicamide, and 0.15% sodium hyaluronate. The bottles were numbered on their bases (in a way that the volunteer could not see), according to the color and the substance. A paper with the number and name of the substance was offered to the volunteer, so that memorization was not necessary. Only the person in charge of applying the test knew the number and the substance of each bottle presented. The participants were then requested to inform the number corresponding to the substance of each bottle. The score of correct answers of each participant was transferred to the Microsoft Excel 2002 electronic spreadsheet and submitted to statistical tests.

## RESULTS

The volunteers’ level of education ranged from incomplete junior school to complete graduate courses. Sixteen of them were males (48%) and 18 were females (52%), age range 21 to 87 years. The index of correct answers in the group of colored eye-drops was 88%, and in the group of transparent eye-drops, 24%.

## DISCUSSION

Eye-drops from several companies were assessed during the study and it was perceived that substances with different indications have very similar flasks, which increases the risk of confusion during the application of the drops. This confusion could be made by the patients themselves and by the persons who apply the medication. The use of wrong substances, besides not causing the desired effect of treatment, could lead to severe side effects, such as ocular burns, when there is misunderstanding with systemic drugs administered in drops.^(^ ^5^ ^)^

Eye-drops of different colors can serve as an alert, in case patients or their caregivers handle the bottle incorrectly. At this moment, change can be made of the medication, or another immediate action may be taken in case of incorrect usage.

The use of colors in eye-drops could be helpful; it is, however, necessary to consider the risk of using natural or artificial pigments, since it is possible that pharmacodynamic and pharmacokinetic changes be induced by such substances. For this reason, the dyes adopted need to be very carefully selected and studied, in order to guarantee that there will be no damage to the ocular surface or any modification in the active ingredient of the eye-drops. All substances used in our test are currently available in Brazil. New medications, with colors that are more easily identifiable, could be produced with this objective.

One limitation of this study was the proximity with which one normally uses eye-drops for patients who utilize their own eye-drops, since the distance is small, and the focus ends up impaired, hindering the correct identification of colors. In this case, identification would be better performed by the caregiver when administering the eye-drop to the patient, who could observe the drop from a more adequate distance. Additionally, appropriate lighting is necessary for color determination.

In our assay, the different colors helped people with intact vision and at a distance at which a good focus could be maintained to better identify the medications. Other methods, such as flexible sleeves with distinct textures and scents, can be used on the bottles of eye-drops to help identification when treating patients with permanently or even temporarily compromised vision, such as in postoperative periods.^(^ ^6^ ^)^

## CONCLUSION

Coloring of the substance can be a factor that aids identification, especially when at an adequate distance to maintain focus. Further studies should be conducted to determine which dyes could be used with no loss in pharmacokinetics of the drugs, and devoid of toxicity for the surface of the eye.
